# Puerarin alleviates osteoporosis in rats by targeting the JAK2/STAT3 signaling pathway

**DOI:** 10.17305/bb.2024.10500

**Published:** 2024-12-01

**Authors:** Xinlei Zhao, Jiaxuan Zhou, Yanqing Liu, Jianguo Wang, Youcai Liu, Beiyu Wang, Caiting Han, Shengjie Zhao, Yijun Zhang

**Affiliations:** 1 Baotou Medical College, Inner Mongolia University of Science and Technology, Baotou, The Inner Mongolia Autonomous Region, China; 2 Department of Orthopaedic Surgery, The First Affiliated Hospital of Baotou Medical College, Inner Mongolia University of Science and Technology, Baotou, The Inner Mongolia Autonomous Region, China

**Keywords:** Osteogenesis, puerarin (Pue), postmenopausal osteoporosis (PMOP), Janus kinase 2/signal transducer and activator of transcription 3 (JAK2/STAT3) signaling pathway, bone metabolism, osteoporosis (OP)

## Abstract

Osteoporosis (OP) is a common chronic progressive bone disease that increases fracture risk in postmenopausal women. Research suggests that puerarin (Pue) may be an effective treatment for OP. This study examined the effects and underlying mechanisms of Pue in treating postmenopausal OP (PMOP) in rats. Sprague–Dawley (SD) rats underwent bilateral ovariectomy to simulate PMOP and were then treated with subcutaneous injections of Pue. Bone mineral density (BMD) was measured using a bone densitometer. Micro-CT scans assessed femur bone structure and various parameters were calculated: bone volume fraction (BV/TV), bone surface density (BS/TV), trabecular thickness (Tb.Th), trabecular number (Tb.N), trabecular separation (Tb.Sp), and bone surface area-to-bone volume ratio (BS/BV). Hematoxylin–eosin (HE) staining was employed to observe femoral tissue pathology. Serum levels of bone formation metabolism-related markers—osteocalcin (OC), bone alkaline phosphatase (BALP), and procollagen type I N-terminal propeptide (PINP)—were measured via enzyme-linked immunosorbent assay (ELISA). The protein expression levels of the Janus kinase 2/signal transducer and activator of transcription 3 (JAK2/STAT3) signaling pathway in bone tissue were evaluated using Western blotting assay. The results showed improved bone density and reduced bone loss in rats treated with Pue. There were also significant increases in serum levels of OC and BALP, indicating enhanced osteogenesis. Furthermore, there was a decrease in activation of the JAK2/STAT3 pathway in femoral tissue, suggesting a pathway inhibition. These findings indicate that Pue may combat osteoporosis by promoting osteogenesis and inhibiting activation of the JAK2/STAT3 pathway activation.

## Introduction

Osteoporosis (OP) is a common chronic and progressive bone disease characterized by reduced bone mineral density (BMD), abnormal bone microstructure, and increased bone fragility. This condition leads to a heightened risk of fractures and has a profound impact on human health [1]. An essential factor in OP is the imbalance between bone formation and bone resorption, leading to increased bone resorption and decreased bone formation, ultimately resulting in severe bone loss [2]. 71% of osteoporotic fractures occur in women, particularly in postmenopausal OP (PMOP) [3]. Surveys indicate that more than 200 million women worldwide are affected by OP, resulting in 8.9 million fractures annually. It is projected that the prevalence of PMOP among women over 50 will reach 13.6 million by 2030 [4]. The leading cause of PMOP is reduced estrogen levels [5, 6]. Estrogen deficiency disrupts bone metabolism, leading to PMOP secondary to lost trabecular bone connections and cortical bone [7]. Estrogen replacement therapy is an effective treatment for OP, but it has side effects, including an increased risk of coronary heart disease, breast cancer, and stroke [8, 9]. Drugs like teriparatide and bisphosphonates have side effects, such as hypercalcemia, osteonecrosis of the jaw, and musculoskeletal pain, and are not recommended for long-term use [10, 11]. Therefore, there is an urgent and unmet need for a new PMOP treatment with fewer side effects.

Currently, several studies are being conducted on the use of phytoestrogens as a replacement for estrogen therapy to prevent PMOP. *Radix Pueraria*, a flavonoid-rich leguminous plant, has been an essential natural food source in China for thousands of years. In 2005, the United Nations Food and Agriculture Organization recommended it as the sixth food crop [12]. Puerarin (Pue), its main ingredient, is an extractable phytoestrogen. Because of its similar structure to estrogen, Pue can bind to estrogen receptors and change the cell’s response to hormones. As a phytoestrogen, Pue has weaker estrogen-like effects and fewer toxic side effects than estrogen [13] and has been proven to have an anabolic effect on bones [14]. As a treatment for PMOP, Pue may be a better choice.

Janus kinase 2 (JAK2)/signal transducer and activator of transcription 3 (STAT3) signaling is crucial in disease progression [15], mainly playing a key role in the occurrence and development of OP [16]. JAK2/STAT3 pathway activation can accelerate the development of PMOP [17]. Wu et al. [18] demonstrated that by inhibiting the JAK2/STAT3 signaling pathway, it is possible to prevent the senescence of bone marrow mesenchymal stem cells in osteoporotic rats, avoid bone loss due to estrogen deficiency, and promote osteogenesis while inhibiting osteoclast formation. Based on these findings, our team hypothesizes that Pue may influence osteogenesis or osteoclast genesis through the JAK2/STAT3 pathway, thereby impacting the occurrence of OP. In this study, a rat model of PMOP was created by performing bilateral ovariectomy (OVX) to replicate the condition’s clinical features [19]. We observed the therapeutic effect of Pue on PMOP and related mechanisms. The research results may inform future clinical applications of Pue as a PMOP treatment.

## Materials and methods

### Animal experiments

We obtained 36 specific pathogen-free female Sprague–Dawley (SD) rats from Beijing Sibeifu Biotechnology Co., Ltd. The rats were eight weeks old, weighed 200 ± 20 g, and were housed in the Animal Experiment Center of Baotou Medical College. They were kept in individually ventilated cages with four rats per cage. The environment was carefully controlled for temperature (20 ^∘^C–25 ^∘^C), humidity (45%–55%), and noise, with 12 h of daily light exposure. The rats were provided with standard feed and sterile water, and their bedding was changed 2–3 times per week.

After one week of adaptive feeding, the rats were randomly divided into sham (*n* ═ 10) and ovariectomize (OVX) groups (*n* ═ 26 rats). Prior to surgery, the rats were fasted for 12 h and the surgical instruments were sterilized using high pressure. The rats were athetized by intraperitoneal injection of 2% pentobarbital sodium (Tianjin Fuchen Chemical Reagent Company, China; batch 20220120)at 30 mg/kg. The rats’ backs were then shaved and disinfected, and a longitudinal incision was made in the middle to access the ovaries. In the OVX group, the ovaries were completely removed, while in the SHAM group, only a small portion of tissue was excised. The incision was then closed and the area was disinfected, and sodium penicillin (Hebei Chengshengtang Animal Pharmaceutical Company, China; 22062601) was injected intraperitoneally for seven days to prevent infection. Four weeks post-surgery, two rats were randomly chosen from both the SHAM and OVX groups for the purpose of measuring lumbar vertebra and femur BMD, as well as observation of femur tissue and performing a HE stain to confirm successful modeling (Ebiogo Company, China; batches B006, B005). Upon successful modeling, the OVX group was divided into the PMOP group, Pue-low-dose (Pue-L), and Pue-high-dose (Pue-H) groups (eight rats per group) and were injected with Pue (Approval number: National Medical Product Approval Number H20020392; Manufacturer: Zhejiang Zhenyuan Pharmaceutical Company, China; 220402; 0.2 g/vial; Purity: >99%) for an 8-week intervention period.

Fully dissolve 0.2 g of Pue for injection with 5 mL of 0.9% sodium chloride solution to prepare the Pue injection with a 40 mg/mL concentration. Prepare it when needed. Based on equivalent conversion, the Pue-L and Pue-H groups received 1 mL/kg and 2 mL/kg for Pue injection, respectively, administered subcutaneously once daily. The SHAM and PMOP groups received an equal volume of normal saline. After eight weeks, BMD was measured and serum and bilateral femurs were collected for further examination.

### BMD measurement

After the rats were anesthetized, they were positioned face-down on the examination table. The lumbar spine and femoral regions of the rats were chosen for measurement. The BMD (g/cm^2^) of their lumbar spine and femoral regions was measured using a dual-energy X-ray absorptiometry bone densitometer (Medilink MEDIX DR, France) of each group of rats.

### Micro-CT analysis

The femurs were prepared and transferred to a high-resolution in vivo micro-CT scanner (Pingsheng Technology Co., Ltd., China). The scans covered the entire length of the femur, from the femoral head to the femoral condyle. The scan settings included a spatial resolution of ≤ 7.5 µm, X-ray energy 90 KV tube voltage, 0.05 mA tube current, 20 frames per second, a total of 4000 frames, and 3600 frames per rotation. The raw data was then analyzed with Avatar software (version 2.0.10.3). A region of interest was selected at a height of 1 mm above the growth plate of the distal femur. The study parameters included bone volume fraction (BV/TV), bone surface area/bone volume (BS/BV), bone surface area density (BS/TV), trabecular thickness (Tb.Th), trabecular number (Tb.N), and trabecular separation (Tb.Sp).

### Hematoxylin and eosin (HE) staining experiment

The slides were dried in an oven at 66 ^∘^C for 20–30 min, preparing the wax film for subsequent experiments. They were then soaked in xylene three times for 5 min each and in a concentration gradient of ethanol three times for 3 min each. Afterward, they were submerged in a beaker and rinsed slowly with running water to completely remove the ethanol until they became clean and transparent.

Next, the slides were stained with hematoxylin for 2–5 min, rinsed with running water, differentiated with 1% hydrochloric acid alcohol for a few seconds, and rinsed again. They were then blued with a saturated lithium carbonate solution for a few seconds and rinsed. The slides underwent dehydration in 95% ethanol for 2 min, then stained with eosin for a few seconds to get the desired color. Finally, they were soaked in 95% ethanol twice for a total of 5–10 s, 100% ethanol twice for 1 min each, and finally in phenol–xylene and two changes of xylene for 2 min each, to complete the staining process. The slides were mounted with neutral balsam and observed using a microscope.

### Enzyme-linked immunosorbent assay (ELISA)

Blood was taken from the abdominal aorta and spun at a centrifugal radius of 10 cm and a rotational speed of 3000 r/min for 10 min to obtain serum supernatant. Diluted samples were then added to sample wells, with separate blank and standard wells included. The plate was sealed and placed in a constant temperature box. A washing solution, biotinylated antibody, and enzyme conjugate were then added, followed by incubation and washing steps. A light-protected reaction was initiated with TMB substrate solution, and any color changes were recorded in the standard wells. To stop the reaction, sulfuric acid was added, and the absorbance of each well was measured using a microplate reader.

Finally, the sample concentrations were calculated based on the standard curve. Bone alkaline phosphatase (BALP) kits (Manufacturer: Jiangsu Enzyme Immunoassay Company, China; batch MM-0436R2), OC and procollagen type I n-terminal propeptide (PINP) kits (Manufacturer: Youersheng Company, China; batch SEA471Ra, CEA957Ra) were used to measure the concentrations of the samples.

### Western blotting

Femur tissues stored at −80 ^∘^C were removed, and GAPDH was used as an internal reference. The tissues were cut into small pieces, lysed with lysis buffer, and centrifuged to collect the supernatant. The protein concentration was determined using a BCA kit. The protein solution was mixed with the sample buffer, denatured by boiling it in a water bath and stored at −20 ^∘^C. Next, a separating gel was prepared and placed in an electrophoresis tank for electrophoresis until the bromophenol blue reached approximately 1 cm from the bottom. A PVDF membrane was activated with methanol, placed on the separating gel, and transferred under constant current conditions. After transfer, the PVDF membrane was blocked with milk and incubated with diluted primary antibodies. The primary antibodies were removed, and the membrane was washed with TBST to remove unbound antibodies. Subsequently, diluted secondary antibodies were added for an immune reaction, followed by another wash. The PVDF membrane was removed and excess liquid was blotted off before adding ECL luminescent solution for a chemiluminescent reaction. The membrane was then exposed in a chemiluminescence instrument and the results were saved. The JAK2 antibody (GB11325), p-JAK2 antibody (GB114585), STAT3 antibody (GB11176), p-STAT3 antibody (GB150001), and IgG antibody (GB23303) were purchased from the Servicebio Company.

### Ethical statement

The research was approved by the First Affiliated Hospital of Baotou Medical College, (approval number 2022016) and conducted according to the guidelines and protocols for animal care and protection.

### Statistical analysis

Statistical analysis and graphing were performed using SPSS 27.0 and GraphPad Prism 9.0. All experiments were conducted at least three times. The data is presented as the mean ± standard deviation (SD). For comparisons among multiple groups satisfying the normal distribution and homogeneity of variance, one-way ANOVA was used. The Kruskal–Wallis H test was used for data that did not satisfy the normal distribution and homogeneity of variance. A *P* value less than 0.05 was considered statistically significant.

## Results

### Establishment of the osteoporosis model

**Figure 1.**
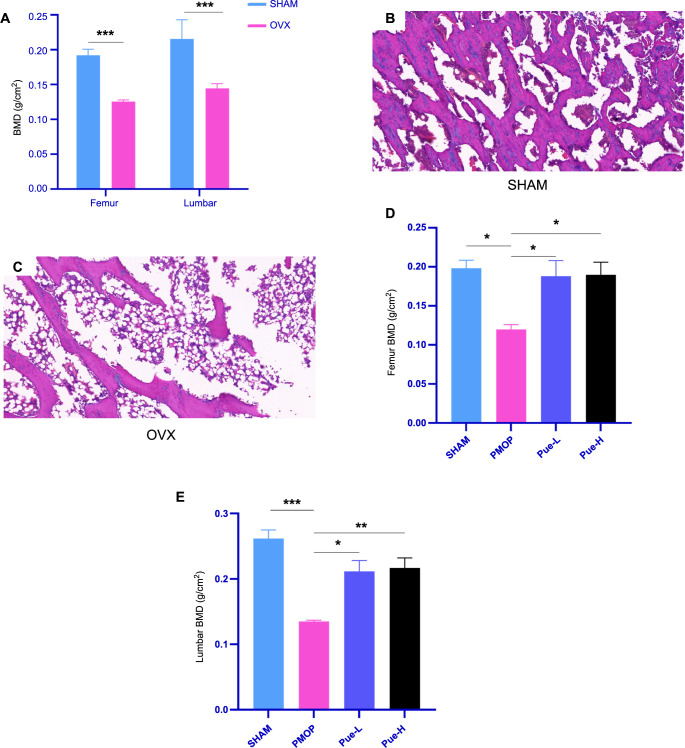
**Effect of high-dose puerarin on BMD and histological changes in ovariectomized rats.** Pue can increase BMD in rats. (A) The femur and lumbar vertebrae BMD in rats four weeks post-surgery; Femoral tissue from rats in the SHAM (B) and OVX (C) groups, observed by HE staining four weeks post-surgery (5x); femur (D) and lumbar vertebrae (E) BMD in SHAM and OVX rats after eight weeks of treatment. Data are presented as mean ± SD (*n* ═ 8). *P < 0.05, **P < 0.01, ***P < 0.001, ns indicates *P* > 0.05. OVX: Ovariectomized; Pue: Puerarin; Pue-L: Low-dose puerarin group; Pue-H: High-dose puerarin group; BMD: Bone mineral density; HE: Hematoxylin–eosin staining; PMOP: Postmenopausal osteoporosis.

Four weeks after the operation, the BMD results showed that—compared with the SHAM group—the lumbar vertebrae and femur BMD in the OVX group decreased significantly (*P* < 0.001) (Figure 1A). HE staining results showed that compared with the SHAM group, the femur trabeculae in the OVX group appeared thinner; some were even broken (Figure 1B and 1C). Combined with the above results, the PMOP rat model was successfully established.

### BMD

After 12 weeks of the operation, the BMDs of the femur and lumbar vertebrae were measured in each group of rats. The BMD of the femur and lumbar vertebrae in the PMOP group was found to be significantly reduced compared to the SHAM group (*P* < 0.05) (Figure 1D and 1E). However, the Pue-L and Pue-H groups showed a significant increase in BMD of both femur and lumbar vertebrae when compared to the PMOP group (*P* < 0.05) (Figure 1D and 1E). This suggests that Pue can enhance bone density in osteoporotic rats. There were no noteworthy differences in lumbar vertebrae BMD between the Pue-L and Pue-H groups compared to the SHAM group. Furthermore, there were no significant differences between the Pue-L and Pue-H groups (*P* > 0.05).

### Micro-CT analysis

**Figure 2.**
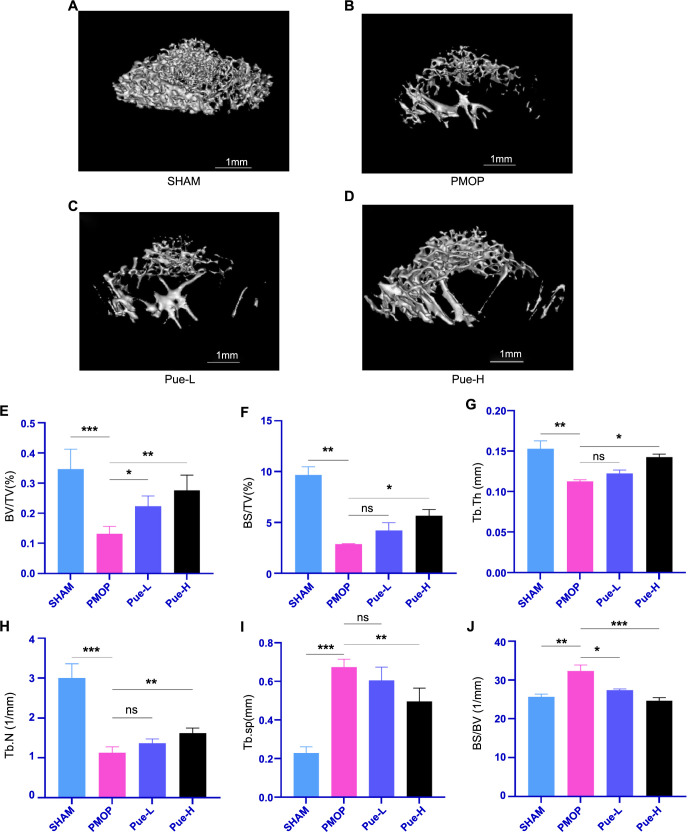
**Pue improves bone loss in PMOP Rats: Micro-CT three-dimensional images and bone microstructure parameters.** (A) SHAM, (B) PMOP, (C) Pue-L, (D) Pue-H, (E) BV/TV, (F) BS/TV, (G) Tb.Th, (H) Tb.N, (I) Tb.Sp, (J) BS/BV. Data are expressed as mean ± SD (*n* ═ 8). *P < 0.05, **P < 0.01, ***P < 0.001, ns indicates *P* > 0.05. PMOP: Postmenopausal osteoporosis; BV/TV: Bone volume fraction; BS/TV: Bone surface density; Tb.Th: The average thickness of trabeculae; Tb.N: Trabecular number; Tb.Sp: Trabecular separation; BS/BV: Bone surface/bone volume; Pue-L: Low-dose puerarin group; Pue-H: High-dose puerarin group.

After conducting micro-CT analysis of bone structural features, distinct differences were found among the groups. Three-dimensional images showed that the SHAM group had normal bone microstructure (Figure 2A), while the PMOP group exhibited severe bone microstructural damage. Specifically, the PMOP group had lower trabeculae and bone density in the distal femur than the SHAM group, indicating significantly higher bone loss (Figure 2B). In contrast, the Pue-L and Pue-H groups showed reduced bone loss and alleviated bone structural damage compared to the PMOP group, with the Pue-H group experiencing even less damage (Figure 2C and 2D). Analysis of Micro-CT bone microstructure parameters for each group of rats revealed several key indicators, including BV/TV, BS/BV, BS/TV, and Tb.Th, Tb.N, and Tb.Sp. These indicators are widely used to reflect bone microstructure.

The results presented in Figure 2E–2J demonstrated that, compared to the SHAM group, the PMOP group had significantly lower values for BV/TV, BS/TV, and Tb.Th, and Tb.N (*P* < 0.01), while Tb.Sp and BS/BV were significantly higher (*P* < 0.01). Compared to the PMOP group, both the Pue-L and Pue-H groups showed increased BV/TV and decreased BS/BV, with statistically significant differences (*P* < 0.05). Additionally, the Pue-H group exhibited significantly higher BS/TV, Tb.Th, and Tb.N, as well as lower Tb.Sp compared to the PMOP group (*P* < 0.05). Although the Pue-L group improved compared to the PMOP group, the differences were insignificant (*P* > 0.05). Overall, the results suggest that the PMOP group experienced significant bone structural damage and loss, while Pue’s treatment improved bone structure and reduced bone loss in PMOP rats.

### Pue can improve trabecular bone structure and quantity

**Figure 3.**
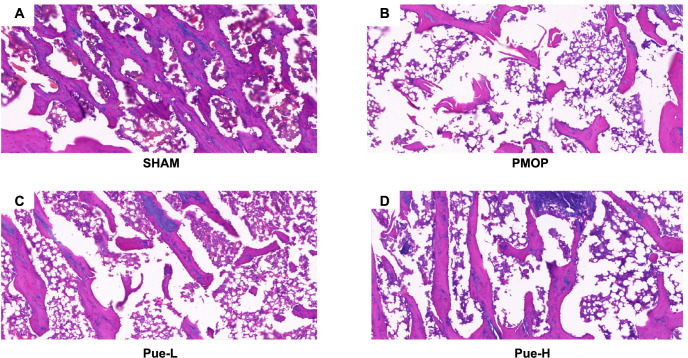
**Pue increases trabecular bone number as observed by HE staining in rat femur tissues.** (A) The SHAM group features normal trabeculae; note the strong and tightly connected structure; (B) the PMOP group features fragile trabeculae that are thinner, shorter, and sparser; note the fragments indicating breakage; the trabeculae in both the Pue-L (C) and Pue-H (D) groups improved in quantity, density, and connection structure, with thicker trabeculae and tighter connections (5x). HE: Hematoxylin – eosin; PMOP: Postmenopausal osteoporosis; Pue-L: Low-dose puerarin group; Pue-H: High-dose puerarin group.

After preparing HE stains of rat femurs, distinct differences in bone histopathology were observed among the various groups. The SHAM group displayed a robust and consistent cortex of compact bone, with closely packed bone cells and interconnected trabeculae (Figure 3A). Conversely, in the PMOP group, the trabeculae appeared thinner with significant fragmentation (Figure 3B). However, the Pue-L (Figure 3C) and Pue-H groups (Figure 3D), showed signs of recovery in the number, density, and structural connectivity of trabeculae compared to the PMOP group, suggesting potential enhancement in bone histopathology.

### Pue promotes osteogenesis in PMOP rats

**Figure 4.**
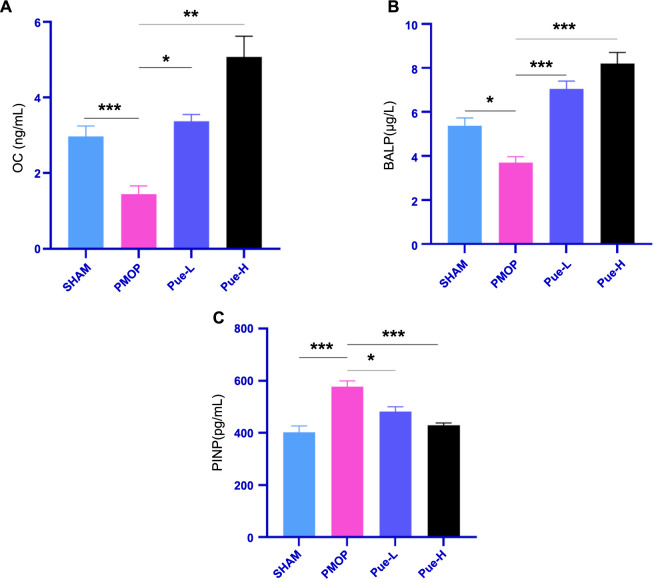
**Pue promotes osteogenesis.** (A) OC, (B) BALP, (C) PINP. Data are expressed as mean ± SD (*n* ═ 8). *P < 0.05, **P < 0.01, ***P < 0.001, ns indicates *P* > 0.05. BALP: Bone alkaline phosphatase; OC: Osteocalcin; PINP: Procollagen type I N-terminal propeptide; SD: Standard deviation; Pue-L: Low-dose puerarin group; Pue-H: High-dose puerarin group; PMOP: Postmenopausal osteoporosis.

According to the results of the ELISA method used to detect osteogenesis-related bone metabolism markers, it was observed that the levels of OC and BALP in the PMOP group were lower compared to the SHAM group. This increase was also observed in the Pue-L and Pue-H groups, indicating that Pue can promote osteogenesis. All differences were statistically significant (*P* < 0.05), as shown in Figure 4A and 4B. Additionally, it was found that the serum PINP levels were higher in the PMOP group than in the SHAM group. Nevertheless, these levels were lower in both the Pue-L and Pue-H groups when compared to the PMOP group. These differences were also statistically significant (*P* < 0.05), as shown in Figure 4C.

### Pue inhibits the JAK2/STAT3 signaling pathway

**Figure 5.**
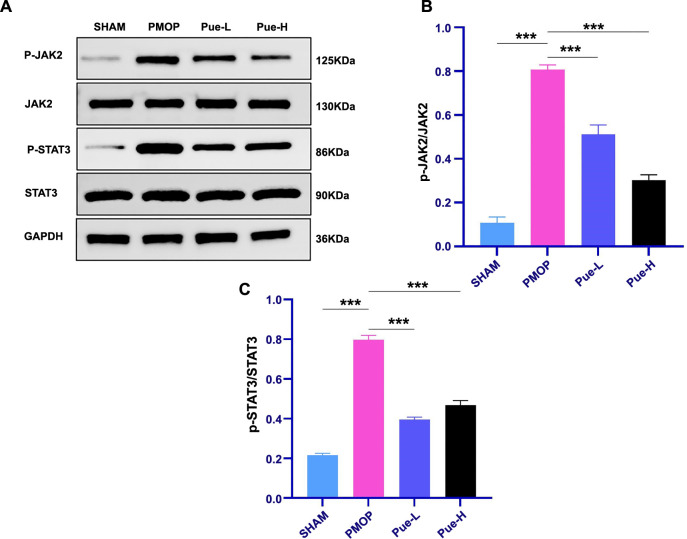
**Pue inhibits activation of the JAK2/STAT3 signaling pathway.** (A–C) Western blotting reveals protein levels of JAK2, p-JAK2, STAT3, and p-STAT3 in the femurs of rats from the SHAM, PMOP, Pue-L, and Pue-H groups. Data are represented as mean ± SD (*n* ═ 8). *P < 0.05, **P < 0.01, ***P < 0.001, ns indicates *P* > 0.05. JAK2/STAT3: Janus kinase 2/signal transducer and activator of transcription 3; SD: Standard deviation; Pue-L: Low-dose puerarin group; Pue-H: High-dose puerarin group; PMOP: Postmenopausal osteoporosis.

Western blot analysis evaluated the expression levels of proteins linked to the JAK2/STAT3 pathway (Figure 5A). The results of this analysis demonstrated that the PMOP group had significantly higher levels of phosphorylated JAK2 (p-JAK2) and phosphorylated STAT3 (p-STAT3) proteins compared to the SHAM group (*P* < 0.001).

**Figure 6.**
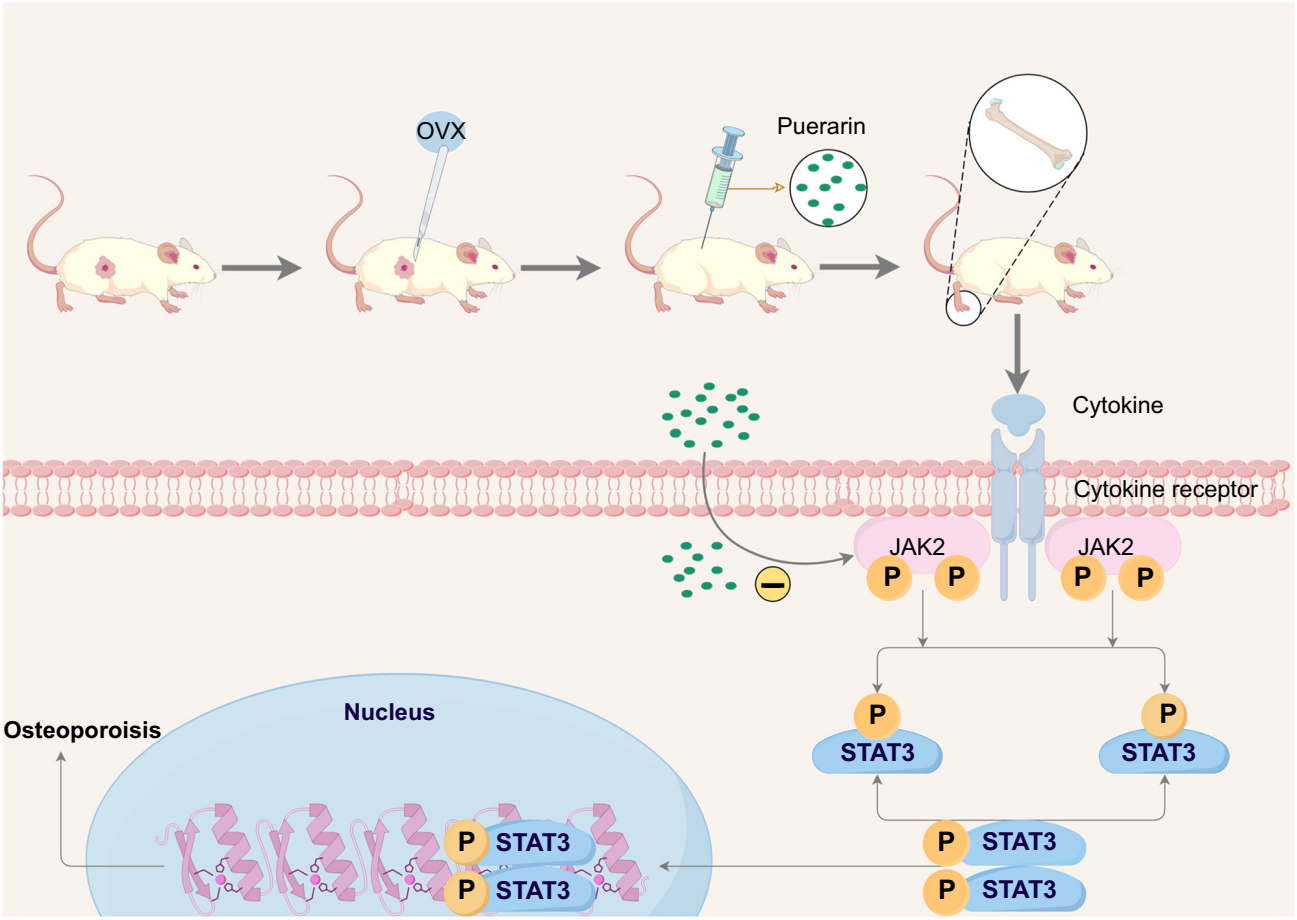
**Schematic diagram of the mechanism of Pue inhibiting JAK2/STAT3 signaling pathway.** Cytokines bind to specific receptors on the cell membrane, activating JAK2. Activated JAK2 phosphorylates the receptors and activates the phosphorylation of STAT3. Phosphorylated STAT3 dimerizes and translocates into the nucleus, where it binds to specific regulatory regions of the DNA sequence to regulate the expression of target genes. Our results show that Pue may inhibit the JAK2/STAT3 signaling pathway activation. Pue: Puerarin; JAK2/STAT3: Janus kinase 2/signal transducer and activator of transcription 3.

This indicates that the JAK2/STAT3 pathway was activated in the rats of the PMOP group. However, in the Pue-treated groups, particularly the Pue-L and Pue-H groups, there was a notable decrease in the expression levels of p-JAK2 and p-STAT3 proteins compared to the PMOP group (*P* < 0.001) (Figure 5B and 5C). These results suggest that Pue effectively inhibits the JAK2/STAT3 signaling pathway.

## Discussion

OP is a systemic metabolic bone disease characterized by bone mass reduction and microenvironment degradation of bone tissue [20]. It is usually undetected before the occurrence of fractures and represents a significant burden for the aging society, becoming a public health issue that must be taken seriously [21]. Bone mass is stable in healthy premenopausal women but begins to decline at an annual rate of approximately 2% for 5–10 years, starting 1–3 years before menopause. Even after this period of rapid bone loss, bone density continues to decline at a rate of 0.5% per year [22]. Estrogen deficiency following natural ovarian aging is the primary cause of PMOP. Decreased estrogen levels during menopause increase the RANKL expression, activating osteoclasts. Osteoblasts cannot build bone at a rate that matches the highly active osteoclasts, thus accelerated bone loss [23]. Currently, the prevention and treatment of OP rely on metabolic modulators but the safety and effectiveness of long-term use are still concerns [1]. Therefore, searching for and identifying new, effective, and safe treatment methods for OP is important.

Plant estrogens are biologically active plant compounds, with estrogens, isoflavones, flavonoids, coumarins, and lignans being the main categories of plant estrogens [24]. Pue, a plant estrogen extracted from kudzu, possesses an isoflavone structure similar to estrogens, allowing it to bind to estrogen receptors and alter cellular responses to hormones [12, 25–27]. Studies have demonstrated that Pue has anti-osteoporotic effects both in vitro and in vivo [28, 29]. It can prevent and treat PMOP by inhibiting osteoclast differentiation through the OPG/RANK/RANKL signaling pathway, thus narrowing the gap in bone metabolism between osteoblasts and osteoclasts [30]. It has been reported that Pue can stimulate bone formation and activate the PI3K/Akt pathway, which regulates the proliferation of osteoblasts in rat calvaria [31]. Studies have confirmed that Pue, a natural compound, can enhance bone formation by osteoblasts and reduce bone resorption by osteoclasts in rats [32, 33]. This study established a rat model of PMOP, and different Pue doses were administered as an intervention. The experimental results revealed that Pue significantly increased BMD, reduced bone loss, and improved bone microstructure in PMOP rats, especially in the high-dose group. These findings suggest that Pue is an effective treatment for PMOP.

Normal bone tissue is in a continuous and dynamic process of bone resorption and formation, known as bone turnover [34, 35]. Bone metabolism indicators refer to relevantions, molecules, and regulatory hormones released into the blood or excreted in the urine during bone turnover. These indicators can represent the activity of osteoblasts or osteoclasts and reflect the rate of bone formation or resorption [36]. The behavior of osteoblasts can be represented by certain markers such as PINP, BALP, and OC [37]. OC is a bone-specific protein that weighs 58 kDa and is made up of 49 amino acids [38]. It is secreted only by osteoblasts, and its blood concentration indicates the bone formation rate [39]. It plays a crucial role in osteoblast function and bone mineralization, making it a specific indicator for bone formation [40]. Osteoblasts also secrete BALP and help in the propagation of bone mineralization. It contributes up to half the total alkaline phosphatase activity in adult blood [41]. PINP is a trimeric peptide with a molecular weight of 35,000 kDa. It exists in the blood as a monomeric degradation product that can be recognized by some immunoassay methods [42]. Serum BALP and PINP concentrations reflect static and dynamic histomorphometric indicators of bone formation [43]. Studies have indicated elevated bone formation markers were observed in the OVX group compared to sham-operated rats [44].

Previous studies investigating Pue’s osteogenic and osteoclastogenic mechanisms in OP have yielded inconsistent results. Some studies have shown that Pue can promote bone formation in rat osteoblasts and reduce bone resorption in osteoclasts [32, 33]. Other experimental findings suggest that Pue treatment can promote osteogenic differentiation of bone marrow mesenchymal stem cells [13]. They may also enhance bone formation by promoting osteoblast differentiation, such as upregulation of alkaline phosphatase mRNA expression [45]. However, Cao [46] discovered that Pue reduces bone loss in OVX-induced osteoporotic mice by inhibiting the bone-resorbing activity of osteoclasts. Meanwhile, Qiu et al. [47] confirmed that Pue prevents bone loss in OVX rats by inhibiting osteoclast activation and bone resorption. Levels of BALP and OC were considerably lower in the PMOP group than in the SHAM group. After receiving Pue treatment, there was a significant increase in BALP and OC levels, indicating improved osteoblast activity and osteogenesis in rats.

However, serum PINP levels were significantly higher in the PMOP group compared to the SHAM group, consistent with previous experimental results. Studies have shown that estrogen deficiency can increase RANKL levels, which is associated with increased bone turnover and high expression of bone resorption cytokines [44, 48]. Previous studies have indicated that Pue can hinder the formation of osteoclasts induced by RANKL by restraining the increase in RANKL during autophagy of osteoclast precursors. Pue is a member of the phytoestrogen compound class, which has a similar structure to estrogen conformation and can bind to estrogen receptors, potentially reducing bone formation markers. However, further validation and exploration are necessary to understand its mechanisms related to osteoclastogenesis.

The JAK/STAT pathway is an intracellular signal transduction pathway expressed widely and involved in various functions, including cell proliferation, differentiation, apoptosis, and immune regulation [49]. Mammals have four members of the JAK family (JAK1-3 and TYK2) and seven of the STAT protein (STAT1-4, STAT5A/B, and STAT6). These proteins combine in different ways to respond to specific cytokines, ensuring a high level of specificity [50]. After a cytokine or growth factor signal binds to its receptor, JAK tyrosine kinases are activated, and then JAK and downstream signaling molecules STAT phosphorylate each other [51]. Among them, the JAK2/STAT3 signaling pathway is crucial in the pathogenesis of diseases [15]. JAK2 tyrosine kinases are typically activated by cytokine or growth factor signaling upstream, and JAK2 phosphorylates the downstream signaling molecule STAT3 [52]. STAT3 is crucial for cell differentiation, growth, and survival. Activation of STAT3 is necessary for osteoblasts to induce RANKL and form osteoclasts [53]. The JAK2/STAT3 pathway activation enhances osteoclast differentiation by promoting RANKL expression in bone cells [54].

The activation of the JAK2/STAT3 signaling pathway has been found to mediate osteoclastogenesis of osteoclast precursors by RANKL [55]. Inhibition of this pathway using the STAT3 inhibitor, Stattic, has demonstrated the ability to suppress osteoclastogenesis in vitro and prevent bone loss in vivo [56]. Another study has shown that naringin can promote the proliferation and osteogenic differentiation of bone mesenchymal stem cells by inhibiting the JAK2/STAT3 pathway, thereby exhibiting an anti-osteoporotic effect [57]. In our study, higher levels of p-JAK2 and p-STAT3 proteins were observed in the PMOP group compared to the SHAM group, indicating increased pathway activity. However, treatment effectively reduced the expression levels and demonstrated the ability to inhibit the JAK2/STAT3 signaling pathway, thereby exhibiting a therapeutic effect on OP.

Although the study has shown that Pue positively affects bone development, it has not explored the mechanism of osteoclastogenesis. Pue can be effective in treating PMOP, but the relationship between the dose and treatment needs further research. The results suggest that Pue may inhibit the JAK2/STAT3 signaling pathway activation (Figure 6). However, further studies are necessary to investigate other mechanisms, including changes in upstream and downstream factors that activate the pathway.

## Conclusion

Pue has been found to reduce bone loss in rats with PMOP, making it an effective treatment for the condition. Pue’s antiosteoporosis effects are due to its ability to promote osteogenesis and inhibit the activation of the JAK2/STAT3 pathway. This suggests that Pue is a potential new drug for treating PMOP.

## Data Availability

The data supporting this study’s findings are included and will be available from the corresponding author(s) upon reasonable request.
